# Supplementary material for: Perioperative Cetuximab with Cisplatin and 5-Fluorouracil in Esogastric Adenocarcinoma: A Phase II Study
Source: Cancers (Basel). 2023 Apr 6;15(7):2188. doi: 10.3390/cancers15072188 (PMC10093434; doi:10.3390/cancers15072188)
Supplement: Supplementary file 1 [file cancers-15-02188-s001.zip › cancers-2262517-supplementary.pdf]

Table S1: Cumulative doses and dose modifications during neoadjuvant treatment

	N (%)
Median duration of neoadjuvant chemotherapy, months	2.3 [0.03-3.6]
Percentage of administered dose/protocol theoretical dose per treatment on all cycles, median [range]	
Cetuximab	96.9 [4.1-105.0]
Cisplatin	97.9 [16.8-105.0]
5FU bolus	97.0 [16.5-104.9]
5FU continuous	97.8 [16.7-104.8]
Treatment modification	
Cetuximab **(n=362)**	
- no modification	329 (90.9)
- hypersensibility	6 (1.7)
- skin toxicity	2 (0.6)
- haematologic toxicity	0 (0)
- non haematologic toxicity	0 (0)
- prescription error	2 (0.6)
- other	23 (6.4)
Cisplatin **(n=370)**	
- no modification	322 (87.3)
- haematologic toxicity	31 (8.4)
- haematologic toxicity + non haematologic toxicity	7 (1.9)
- non haematologic toxicity	6 (1.6)
- prescription error	0 (0)
- other reason	3 (0.8)
5FU **(n=369)**	
-no modification	314 (85.1)
-haematologic toxicity	35 (9.5)
- haematologic toxicity + non haematologic toxicity	7 (1.9)
- non haematologic toxicity	9 (2.4)
- prescription error	0 (0)
- other	4 (1.1)
Cycle delay	
- At least one delay	36 (55.4)
- Number of cycle delay	44
Reasons for cycle delay	
- Department organization	9 (20.5)
- Department organization + patient's will	1 (2.3)

- Other reasons	9 (20.5)
- Toxicity	23 (52.3)
- Patient convenance	2 (4.5)
Reason for definitive stop of the treatment	
-end of protocolary treatment	57 (89.1)
-investigator's decision	7 (10.9)
-major toxicity	3
-disease progression	1
- intercurrent event	1
- drop out	0
-other reason	2
-patient convenance	0 (0)
-death	0(0)

Table S2: Cross-tabulation of the radiological response obtained by the centralized review and the response given by the centers

	Response given by the centers									
	Partial response		***Stability***		***Progression***		***Not Evaluable***		*Total*	
	N	%	N	%	N	%	N	%	N	%
**Centralized response**										
Partial response	10	58.82	7	41.18	0	0.00	0	0.00	17	100.00
Stability	8	22.86	21	60.00	2	5.71	4	11.43	35	100.00
Progression	0	0.00	3	50.00	2	33.33	1	16.67	6	100.00
Not Evaluable	2	28.57	2	28.57	1	14.29	2	28.57	7	100.00
Total	20	30.77	33	50.77	5	7.69	7	10.77	65	100.00

Table S3: Cumulative doses and dose modifications during adjuvant treatment

	N (%)
Median duration of neoadjuvant chemotherapy	2.4 months [0.03 - 3.9]
Percentage of administered dose/protocol theoretical dose per treatment on all cycles, median [range]	
Cetuximab	97.8 [13.2-117.4]
Cisplatin	76.7 [11.6-104.5]
5FU bolus	80.6 [16.7-104.4]
5FU continu	79.1 [16.7-104.3]
Dose reduction	
Cetuximab	17 (35.4)
Cisplatin	29 (60.4)
5FU	27 (56.3)
Treatment modification	
Cetuximab **(N=239)**	
- no modification	192 (80.3)
- skin toxicity	3 (1.3)
- haematologic toxicity	1 (0.4)
- haematologic + non haematologic toxicity	3 (1.3)

- non heamatologic+ other reason	3 (1.3)
-prescription error	2 (0.8)
-other	35 (14.6)
Cisplatin **(N=236)**	
- no modification	136 (57.6)
- haematologic toxicity	54 (22.9)
- haematologic toxicity + non haematologic toxicity	15 (6.4)
- hamatologic+other reason	4 (1.7)
- non haematologic toxicity	23 (9.7)
- other reason	4 (1.7)
5FU **(N=244)**	
- no modification	134 (54.9)
- haematologic toxicity	46 (18.9)
- haematologic toxicity + non haematologic toxicity	15 (6.1)
- haematologic toxicity + other reason	3 (1.2)
- non haematologic toxicity	31 (12.7)
- non heamatologic toxicity + other reason	1 (0.4)
- other reason	14 (5.7)
Cycle delay (n=48)	29 (60.4)
- At least one delay	
Reasons for cycle delay: (49 cycle delay)	
- Toxicity	29 (59.2)
- Department organization	6 (12.2)
- Department organization + toxicity	1 (2.0)
- Other reasons	7 (14.3)
- Patient convenance	6 (12.2)

Table S4: quality of life

QLQ-C30	Before initiation of neoadjuvant treatment			After neoadjuvant chemotherapy, before surgery			After surgery , before adjuvant treatment			After adjuvant treatment (first questionnaire)		
Score	N	Mean	SD	N	Mean	SD	N	Mean	SD	N	Mean	SD
Global health status	58	69.5	21.0	44	59.5	17.0	33	54.0	19.0	44	65.5	17.9
Physical functionning	60	91.6	13.9	43	82.4	17.3	33	66.5	25.3	44	79.9	16.7
Role functionning	60	88.6	19.5	42	71.0	29.0	33	58.1	30.7	44	65.5	27.0
Emotional functionning	59	78.0	18.3	44	75.7	20.4	32	72.1	21.0	44	80.1	18.5
Cognitive functionning	60	90.8	16.6	43	87.6	14.6	32	84.4	16.9	44	84.5	17.4
Social functionning	59	91.0	15.6	43	72.1	26.2	33	65.2	28.4	44	76.5	25.0
Fatigue	60	25.3	21.8	44	45.8	25.6	33	50.7	27.1	44	42.8	26.4
Nausea/vomiting	60	10.8	21.0	44	20.1	19.9	33	16.7	24.3	44	16.7	21.0
Pain	59	17.8	22.1	44	14.4	17.8	32	38.0	26.5	44	23.5	20.1
Dyspnea	56	14.9	25.4	44	15.9	23.3	32	33.3	30.5	43	28.7	24.8
Insomnia	58	27.0	30.9	44	22.0	26.8	33	32.3	32.8	44	23.5	28.4
Appetite loss	59	19.2	31.1	43	40.3	39.5	33	39.4	34.8	44	36.4	33.6
Constipation	59	14.7	25.0	42	32.5	32.5	31	14.0	25.5	44	15.9	23.3
Diarrhoea	60	5.6	16.4	44	11.4	18.9	33	33.3	31.2	43	23.3	24.7
Financial difficulties	59	10.7	25.1	43	11.6	21.7	32	17.7	28.1	44	16.7	27.4
STO-22	Before initiation of neoadjuvant treatment			After neoadjuvant chemotherapy, before surgery			After surgery , before adjuvant treatment			After adjuvant treatment (first questionnaire)		
Score	N	Mean	SD	N	Mean	SD	N	Mean	SD	N	Mean	SD
Body image	58	5.2	13.7	43	22.5	26.9	33	20.2	28.8	42	15.1	22.3
Dysphagia	60	21.9	24.9	40	19.4	25.8	32	24.7	24.8	42	11.1	16.3
Pain	60	21.6	17.2	41	18.5	19.6	32	23.7	20.9	42	22.6	18.0
Reflux	60	15.4	15.5	42	14.7	16.0	33	17.3	18.5	42	16.3	19.8
Food restrictions	60	21.0	21.7	43	32.0	27.2	32	34.2	19.6	42	24.4	22.3
Anxiety	60	41.3	22.0	43	39.8	25.0	33	39.4	25.6	41	34.1	24.6
Dry mouth	59	12.4	23.1	42	38.9	36.0	33	36.4	33.7	42	25.4	29.3
Trouble with taste	60	11.7	23.6	43	41.9	36.4	31	28.0	32.3	41	25.2	30.5
Hair fall	3	11.1	19.2	18	27.8	32.8	16	25.0	39.4	7	47.6	32.5

SD: standard
